# Role of Steatosis in Preventing Post-hepatectomy Liver Failure After Major Resection: Findings From an Animal Study

**DOI:** 10.1016/j.jceh.2024.102453

**Published:** 2024-11-13

**Authors:** Andrea Lund, Mikkel T. Thomsen, Jakob Kirkegård, Anders R. Knudsen, Kasper J. Andersen, Michelle Meier, Jens R. Nyengaard, Frank V. Mortensen

**Affiliations:** ∗ Department of Surgery, Section for HPB Surgery, Aarhus University Hospital, Aarhus, Denmark; † Department of Clinical Medicine, Aarhus University, Aarhus, Denmark; ‡ Core Center for Molecular Morphology, Section for Stereology and Microscopy, Aarhus University, Denmark; § Department of Pathology, Aarhus University Hospital, Denmark

**Keywords:** 90% partial hepatectomy, stereology, lipid metabolism, hepatic lipid content, liver regeneration

## Abstract

**Background/Aim:**

Post-hepatectomy liver failure (PHLF) and hepatic steatosis are evident shortly after extensive partial hepatectomy (PH) in rodents. This study aimed to extrapolate the protein expression and biological pathways involved in recovering PHLF (rPHLF) and non-recovering PHLF (nrPHLF).

**Methods:**

Rats were randomly assigned to 90% PH or sham surgery. rPHLF was distinguished from nrPHLF using a quantitative scoring system. The sham (n = 6), rPHLF (n = 8), and nrPHLF (n = 13) groups were compared 24 h post-PH. Proteomics was used to assess protein variations and to investigate differentially regulated biological pathways. Stereological methods were used to quantify hepatic lipid content. The plasma triglyceride levels were measured.

**Results:**

rPHLF demonstrated substantial downregulation of proteins involved in lipid metabolism compared to nrPHLF (*P* < 0.001). Several proteins associated with lipogenesis, beta-oxidation, lipolysis, membrane trafficking, and inhibition of cell proliferation were markedly downregulated in rPHLF.

The hepatic lipid proportion was significantly higher for rPHLF (61% of hepatocyte volume, 95% confidence interval [CI]: 48%–82%) than for nrPHLF (32% of hepatocyte volume, 95% CI: 22%–39%). The median lipid volume per hepatocyte in rPHLF was 2815 μm^3^ (95% CI: 2208–3774 μm^3^) and 1759 μm^3^ in nrPHLF (95% CI: 1188–2134 μm^3^). Lipid droplets were not detected in the sham-operated rats. No significant differences in plasma triglyceride levels were found between the groups (*P* > 0.08).

**Conclusion:**

The degree of hepatic steatosis is a promising prognostic indicator for early liver regeneration and nrPHLF onset immediately following extensive PH. Intrahepatic lipid accumulation appears to be linked to the coordinated downregulation of proteins integral to lipid metabolism and cellular transport.

Liver resection remains the gold standard treatment for liver malignancies. The regenerative capacity of the liver allows for partial hepatectomy (PH) of up to 70% of the parenchyma in a single procedure.^1^ Exceeding this limit significantly increases the risk of posthepatectomy liver failure (PHLF), which is associated with high morbidity and mortality.^2^ Exploring the biological processes involved in PHLF is crucial for identifying novel biomarkers of this critical condition. Such biomarkers hold the potential to improve outcomes following extensive liver resection and to guide interventions aimed at managing or preventing PHLF.

Transient steatosis occurs within the first day after extensive PH in rodents,^3^, ^4^, ^5^, ^6^ but data on metabolic changes in regenerating hepatocytes, especially those related to lipid metabolism, are limited. Lipid metabolism is a complex process that involves the liver as an essential depot for lipid uptake, storage, breakdown, and release. This intricate system relies on a diverse array of proteins that serve as catalysts, regulators of gene expression, and vital components of signaling pathways that regulate lipid homeostasis.^7^ Precision in regulatory functions typically limits the accumulation of lipid droplets in the liver. However, factors, such as liver resection and obesity, can induce an imbalance in lipid metabolism, contributing to liver steatosis.

Although preoperative steatosis increases the risk of PHLF after liver resection owing to endoplasmic reticulum stress, mitochondrial dysfunction, impaired autophagy, and inflammation,^8^, ^9^, ^10^ transient steatosis emerging after PH may benefit liver regeneration. These benefits arise from lipids serving as an additional source of energy, acting as building blocks for new cell membranes and potentially functioning as proximal signals to initiate cell proliferation.^11^, ^12^, ^13^, ^14^

We previously identified the rat model of 90% PH as a PHLF model, in which all rats developed biochemical PHLF, but some were able to reverse this condition.^15^ In this study, we investigated the proteins and biological pathways involved in PHLF. We hypothesized that protein expression and pathway regulation differed between recovering PHLF (rPHLF) and non-recovering PHLF (nrPHLF) and that transient steatosis following PH was influenced by the regulation of lipid metabolism.

## MATERIALS AND METHODS

### Ethical Approval

This study was approved by the Danish National Committee for the Protection of Animals used for Scientific Purposes, Copenhagen, Denmark, under the license number 2021-15-0201-00978. The animals were handled in compliance with the Guide for the Care and Use of Laboratory Animals of the US National Institutes of Health,^19^ and the minimum number of animals required to identify a significant difference between groups was utilized. This study adhered to the reporting standards defined in the Animal Research: Reporting of In Vivo Experiments (ARRIVE) guidelines.^20^

### Experimental Design

Male Wistar rats were randomized to either 90% PH or sham surgery (midline laparotomy without liver resection) group. A quantitative scoring system assessing physiological and behavioral parameters was used to distinguish rPHLF from nrPHLF, as described in detail previously.^15^ As nrPHLF became evident 24 h after PH, this timepoint was selected to compare the nrPHLF (n = 13), rPHLF (n = 8), and sham (n = 6) groups.^15^

After euthanasia, the liver remnants were harvested, and blood samples were collected, processed, snap-frozen in liquid nitrogen, and stored at −80 °C until analysis. The anterior caudate lobes were snap-frozen in liquid nitrogen before being stored at −80 °C until liquid chromatography–tandem mass spectrometry (LC-MS/MS) analysis. The posterior caudate lobes were fixed in phosphate-buffered 4% paraformaldehyde before being embedded in paraffin for stereological analysis. Rat liver tissues from a previous 90% PH study^15^ were immersion-fixed in phosphate-buffered 4% paraformaldehyde, followed by 2% glutaraldehyde for transmission electron microscopic analysis.

### Sample Preparation and LC-MS/MS

#### Lysing and Homogenization

Liver tissue masses were measured before being transferred into lysing Matrix D kits for Fastprep-24 5G (Avantor, PA, US). Thereafter, 5 μl of lysis buffer per milligram of wet tissue was added to each sample. The lysis buffer contained 100 mM Tris-HCl, 150 mM NaCl, 5% sodium dodecyl sulfate, 2 mM phenylmethylsulfonyl fluoride, and 2 μM E-64; the pH was adjusted to 8. After adding lysis buffer, the samples were homogenized at 6 m/s for 20 s and immediately placed on ice. Suspensions of 300 μl were taken from the homogenized samples and diluted with 900 μl of lysis buffer in new tubes. The samples were incubated at 95 °C for 5 min and clarified by centrifugation at 20 000×*g* for 10 min at 4 °C. The protein concentration of each sample was determined using the Pierce BCA Protein Assay Kit (Thermo Fisher Scientific, Waltham, MA, US) according to the manufacturer's instructions.

#### Filter-aided Sample Preparation

Volumes containing 100 μg of total protein were taken from each sample and transferred to a 10-kDa filter device (AcroPrep advanced 96-well plates, Pall). Disulfides were reduced by adding 8 M urea and 0.1 M Tris-HCl at pH 8.5 with 25 mM dithiothreitol, and the samples were incubated for 30 min. After disulfide reduction, the samples were centrifuged at 1500×*g* to remove the dithiothreitol solution. Subsequently, they were alkylated for 30 min by adding 8 M urea and 0.1 M Tris-HCl at pH 8.5 with 50 mM iodoacetamide. The iodoacetamide solution was removed by centrifuging and filtering the samples twice with 8 M urea and 0.1 M Tris-HCl at pH 8.5, followed by rinsing twice with 50 mM NH_4_HCO_3_. All samples were digested with 1 μg trypsin (Promega) in 50 μl of NH_4_HCO_3_ for 18 h at 37 °C. The tryptic peptides were collected by centrifuging and sequentially adding and collecting 50 μl of 50 mM NH_4_HCO_3_, 50 μl of 0.5 M NaCl, and 50 μl of 0.1% formic acid. All samples were desalted by micropurification using microcolumns packed with Empore Solid Phase Extraction Disks (3M, MN, US) prepared in-house, as described previously.^16^

#### LC-MS/MS

LC-MS/MS was performed on an EASY-nLC 1200 system (Thermo Fisher Scientific, MA, US) connected to an Orbitrap Eclipse Tribrid Mass Spectrometer (Thermo Fisher Scientific, MA, US). The peptides were dissolved in 0.1% formic acid, injected, trapped, and desalted on a trap column (2 cm × 100 μm inner diameter). The peptides were then eluted from the trap column and separated in a 15-cm analytical column (75 μm inner diameter). Both columns were packed with 3-μm ReproSil-Pur C18-AQ resin (Dr. Maisch, Ammerbuch-Entringen, Germany). The peptides were eluted at a flow rate of 250 nl/min and a 50-min gradient from 5% to 40% phase B (0.1% formic acid and 80% acetonitrile), followed by 10 min of 100% phase B. The MS data were processed using Proteome Discoverer 2.4 (Thermo Fisher Scientific, MA, US). The data were searched for in a rat reference proteome from UniProt using the Sequest HT search engine (*Rattus norvegicus*, one protein sequence per gene, 21 584 sequences). The search parameters were trypsin as the protease, minimum peptide length of six residues, precursor mass tolerance of 10 ppm, and fragment mass tolerance of 0.02 Da. The static modifications were set to carbamidomethyl, and the dynamic modifications were set to oxidation of Met and acetylation of the N-terminus. The search results were used for precursor ion quantification based on the summed abundances and normalized to the total peptide amount. All the raw MS data were manually inspected. Samples with very low intensities in the total ion chromatogram, suggesting suboptimal sample preparation and loading, were excluded from analysis. Considering that the data were normalized to the total ion intensities, samples with very low intensities did not reflect the proteome and were consequently excluded. Precursor quantification required that the precursor be present in at least 40% of the subsamples and that replicate-based resampling be used for imputation or low-abundance resampling in cases of no detected precursors in a treatment group. Owing to the risks associated with imputing data, this imputation was performed only when a precursor was found in 100% of the subsamples of the other treatment groups, suggesting that the missing values in the group in question likely reflected a biological change. Precursors that did not fulfill these criteria were omitted from the downstream analyses.

### Transmission Electron Microscopy

Cubes (2 mm^3^) of fixed liver tissue were processed and embedded in resin (Standard TAAB 812 Resin kit, TAAB Laboratory Equipment Ltd, Berks, UK) using standard transmission electron microscopy protocols. Ultrathin sections were cut using an ultramicrotome (Leica Microsystems, Wetzlar, Germany), and transmission electron microscopy images were obtained using a JEM-1010 electron microscope (JEOL Ltd, Akishima, Tokyo, Japan) with an Orius SC1000 CCD digital camera (Gatan, Inc., Pleasanton, CA, USA).

### Stereological Quantifications

**Figure 1:**
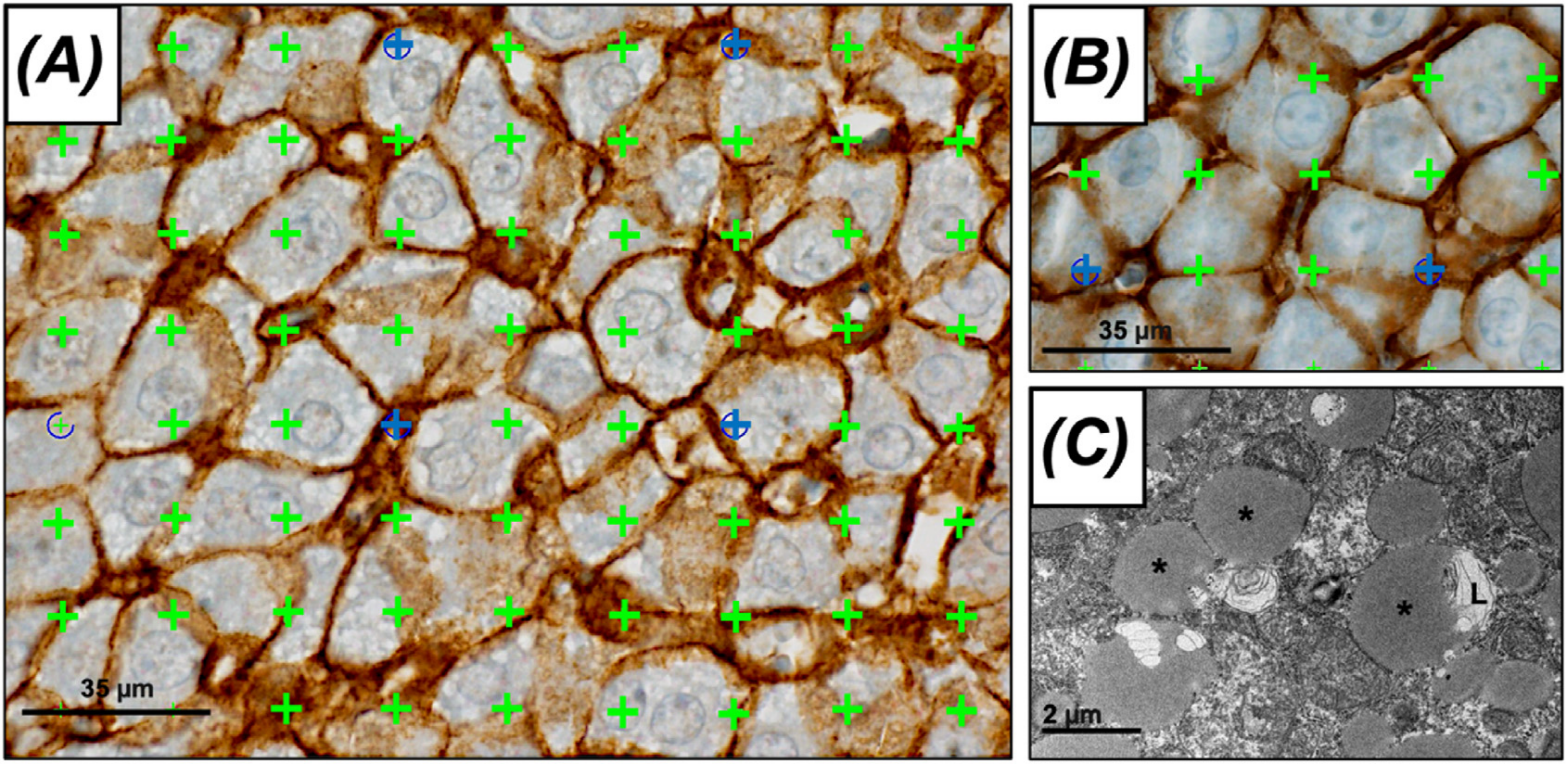
**Lipid content visualized by light microscopy and transmission electron microscopy.** (A) Liver section from the rPHLF group. Ki-67, beta-catenin, and hematoxylin staining. Lipid droplets appear as clear, empty vacuoles. The lipid proportion within a hepatocyte was estimated using a point grid, marked with green and blue cross marks. Points were counted when the upper-right corner of a cross mark intersected with the region of interest. Every cross mark hitting a lipid droplet was tallied, whereas only the blue cross marks that coincided with a hepatocyte were included in the count. (B) Liver section from the sham group showing no visible lipid droplets. (C) An electron microscopy image of a hepatocyte in the rPHLF group with massive lipid droplets (∗), some infiltrated by lysosomes (L). rPHLF, recovering posthepatectomy liver failure.

The posterior caudate lobes were prepared for stereological quantification, as previously described.^17^ A blinded investigator analyzed all sections using an Olympus BX50 microscope, modified for stereology using a motorized stage (Märzhäuser Wetzlar MFD, Wetzlar, Germany) and a digital camera (Olympus DP73, Olympus, Tokyo, Japan) connected to a computer running newCAST version 2020.08.4.9377 software (Visiopharm, Hørsholm, Denmark). A 9 × 4 point grid was used to estimate the lipid droplet content within each hepatocyte. Microscopy was performed using a 60 × oil objective lens (N.A., 1.30). On average, 30 systematically, uniformly, and randomly selected fields of view per animal were used. Validated by electron microscopy, lipid droplets were defined as clear empty vacuoles,^18^ as illustrated in Figure 1. In the rPHLF group, approximately 100 test points intersecting lipid droplets were counted per animal.

The volume of lipid droplets within each hepatocyte, denoted by V(lipid in hepatocyte), was calculated using the following formulas:

$$V_{v}\left(\frac{\text{lipid}}{\text{hepatocyte}}\right)=\frac{\sum P\left(\text{lipid}\right)}{\sum P\left(\text{hepatocyte}\right)}$$

$$V\left(\text{lipid}\;\text{in}\;\text{hepatocyte}\right)=V_{v}\left(\frac{\text{lipid}}{\text{hepatocyte}}\right)\cdot V\left(\text{hepatocyte}\right)$$

where ΣP represents the sum of the test points hitting the lipids and hepatocytes. V(hepatocyte) refers to the mean volume of hepatocytes, which we previously estimated using the isotropic nucleator for both rPHLF (4485 μm^3^) and nrPHLF (5498 μm^3^).^17^ The counting rules are illustrated in Figure 1.

### Statistical Analyses

Group comparisons of immunohistochemical and biochemical variables were performed using analysis of variance, whereas pairwise comparisons were performed using unpaired t-tests. Parametric data are expressed as means and 95% confidence intervals (CIs). Non-parametric immunohistochemical variables were log transformed, analyzed, and then back-transformed before being presented as median values with 95% CIs. Statistical significance was set at *P* < 0.05. Analyses were performed using Stata version 17.0 (StataCorp LLC, College Station, TX, U.S.).

The LC-MS/MS protein expression data were statistically analyzed using Proteome Discoverer 2.4 (Thermo Fisher Scientific, MA, US). Protein ratio calculations were based on pairwise peptide ratios and evaluated using a t-test.

A principal component analysis (PCA) was used to depict clustering patterns between groups (R packages “FactoMineR” v. 2.9 and “factoextra” v. 1.0.7), and volcano plots were used to visualize the differential protein expression results (R package “EnhancedVolcano” v. 1.14.0). The fold-change threshold of interest was set to 1, and the corresponding adjusted *P* values of <0.05 were considered statistically significant. The upregulated and downregulated proteins were queried in the Gene Ontology (GO): Biological Process (BP) and GO: Molecular Function (MF) pathway databases and subjected to pathway enrichment analysis using the R package “clusterProfiler” (v. 4.4.4). The pathway *P* values were adjusted using the Fisher's exact test, and only pathways with an false discovery rate (FDR) <0.05 were considered statistically significant.

A heat map of the 40 most regulated proteins across the groups, based on adjusted *P* values and fold changes, was prepared using the R package “pheatmap” (v. 1.0.12), with the normalized data clustered using Euclidean distance and the complete clustering method as input parameters.

## RESULTS

### Proteomic Profiling and Bioinformatics Analysis

**Figure 2:**
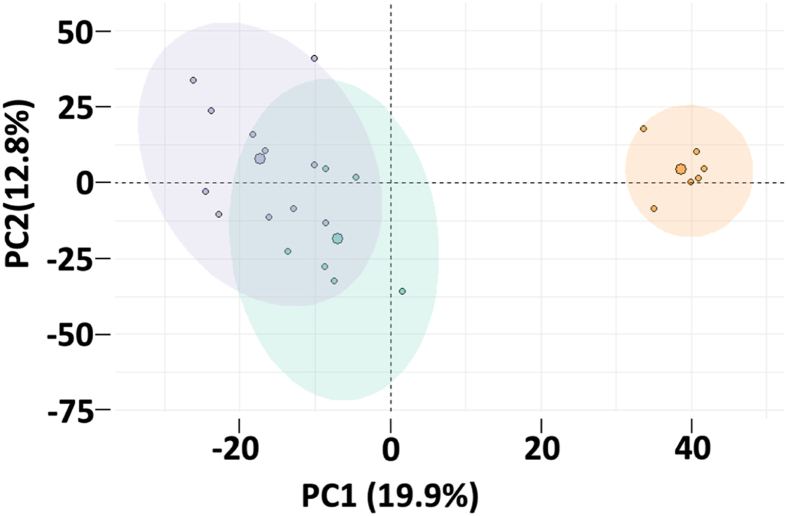
**PCA plot of the proteomic data from the PH and sham groups.** A total of 2865 proteins were included in the analysis. The data were normalized and scaled prior to the analysis. The overall differences between the rPHLF and the nrPHLF groups were smaller than those between the PH groups and the sham group. Green: rPHLF; purple: nrPHLF; orange: sham. nrPHLF, non-recovering posthepatectomy liver failure; rPHLF, recovering posthepatectomy liver failure; PCA, principal component analysis; PH, partial hepatectomy.

A total of 3462 proteins were detected across all samples; however, after filtering the data for low abundance detection, as described earlier, 2865 proteins remained, which were included in the downstream analyses. The PCA plot revealed distinct clustering patterns among the three groups, with the greatest similarities observed between the PH groups (Figure 2).

**Figure 3:**
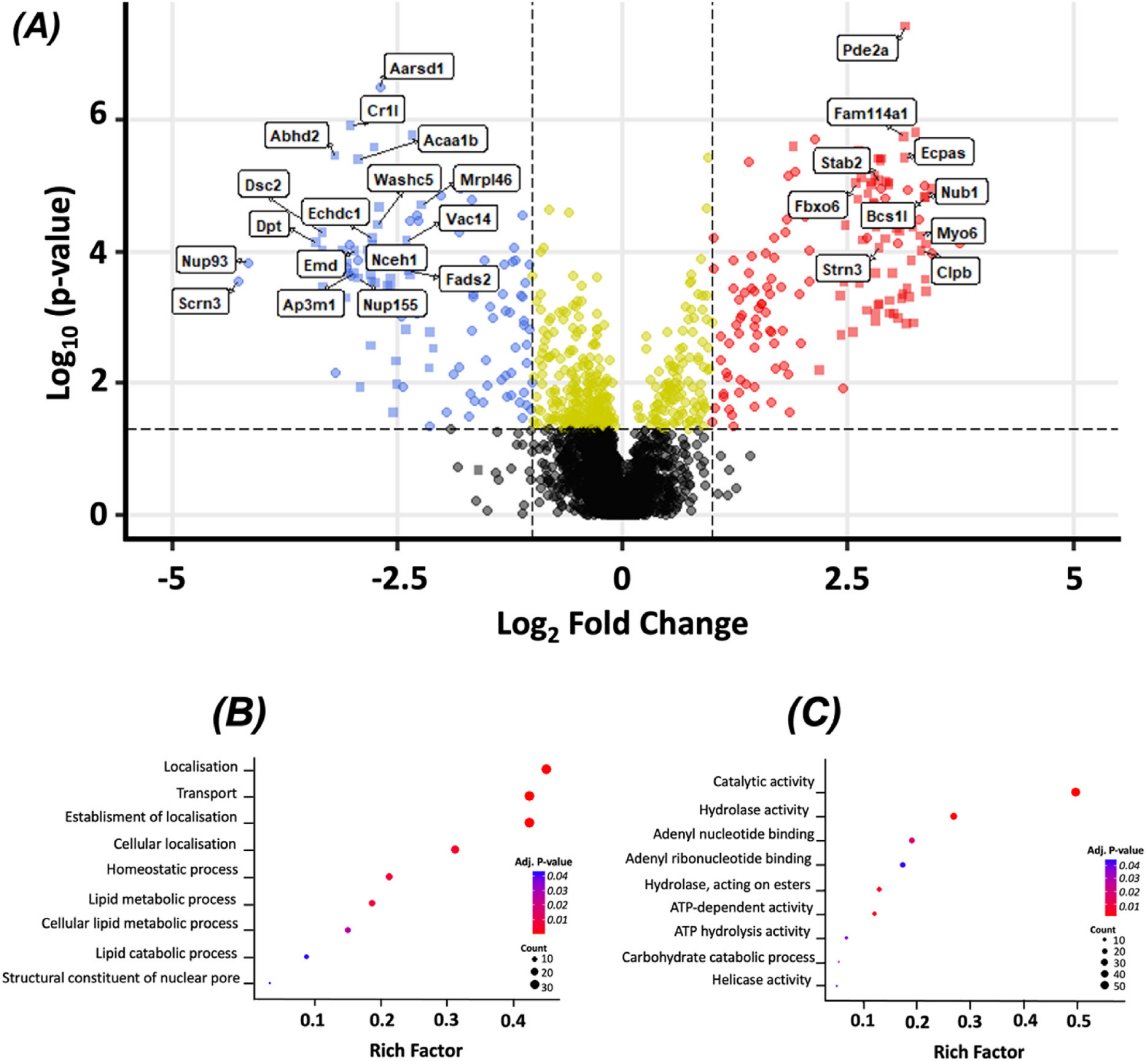
**Differentially expressed proteins in liver samples from rPHLF and nrPHLF.** (A) Volcano plot of all proteins identified in the two groups through proteomic analysis. The gray points denote proteins with statistically nonsignificant changes, the yellow points represent proteins with statistically significant changes smaller than a Log2-fold change of 1, the red points indicate upregulated proteins, and the blue points indicate downregulated proteins in rPHLF compared to nrPHLF. The squares indicate imputed data (proteins not identified in any samples of a group but identified in all samples of the other group). (B) Functional enrichment analysis based on significantly downregulated proteins. (C) Functional enrichment analysis based on upregulated proteins. The bubble plots visualize the significantly downregulated and upregulated GO:BP and GO:MF terms. nrPHLF, non-recovering posthepatectomy liver failure; rPHLF, recovering posthepatectomy liver failure.

Of the 2865 detected proteins, 219 exhibited significant differential expression between rPHLF and nrPHLF. The over-representation analysis revealed that rPHLF showed significant differential regulation of 18 biological pathways, with half of them being downregulated and half of them being upregulated, compared to nrPHLF (Figure 3). Three pathways exclusively featured proteins essential for lipid homeostasis.

To examine the potential regulation of transport mechanisms involved in lipid metabolism, the downregulated proteins associated with the GO:BP categories “Transport” and “Lipid Metabolic Process” were compared. This analysis revealed the presence of four shared proteins, including hepatocyte nuclear factor 4 alpha (HNF4A), which are associated with both intracellular trafficking and very low-density lipoprotein (VLDL) secretion. The remaining proteins associated with the GO:BP category “Lipid Metabolic Process” were implicated in various aspects of lipid metabolism, such as lipolysis, detoxification, and regulation of gene expression (Supplementary Table 1).

**Figure 4:**
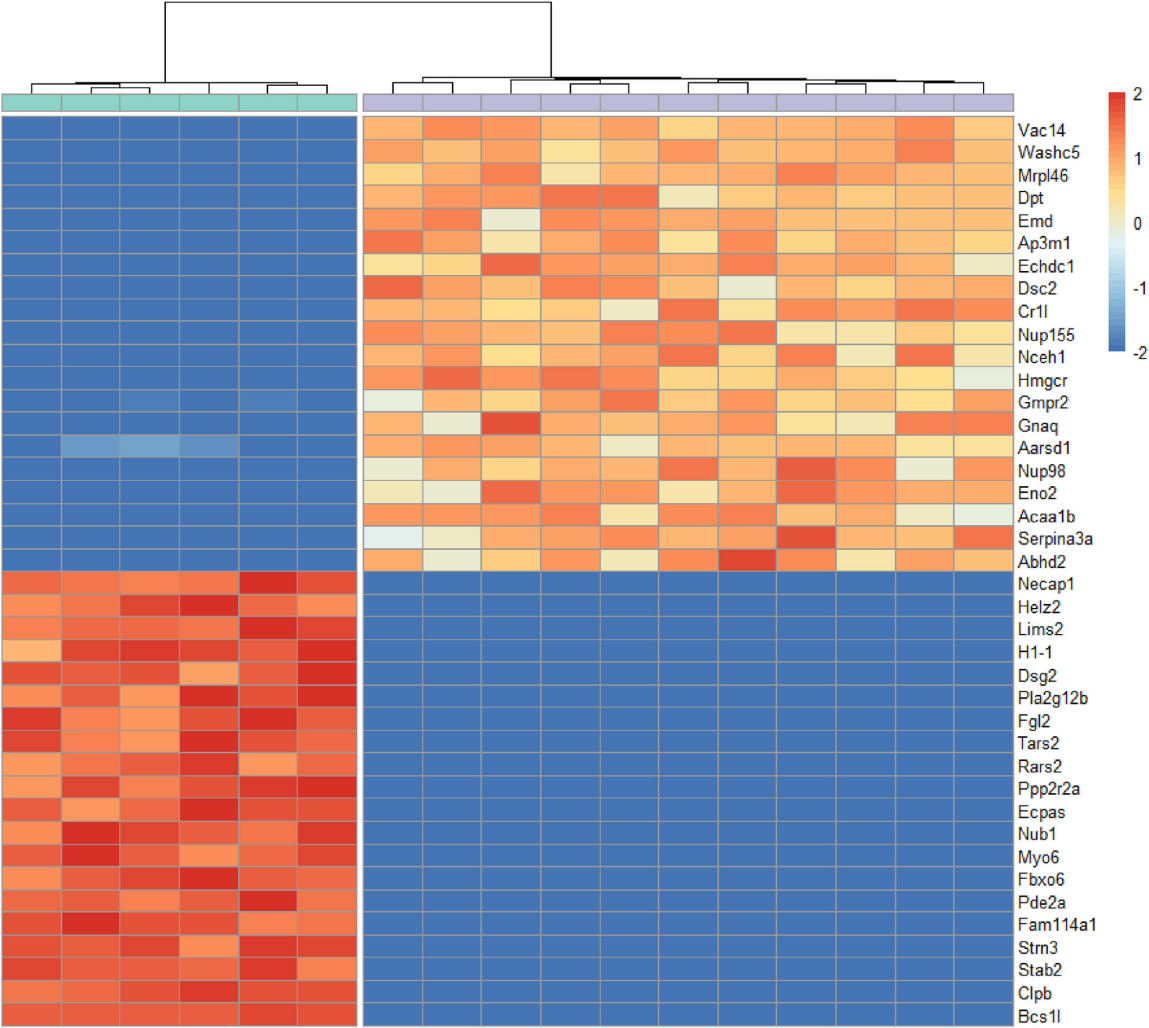
**Heat map representing the top differentially expressed proteins in liver samples from rPHLF and nrPHLF.** Each row corresponds to a specific protein, and each column represents a liver sample. The color intensity within each cell reflects the normalized and scaled abundance of the corresponding protein in the respective sample, with a gradient ranging from blue (low abundance) to red (high abundance). Green: rPHLF; purple: nrPHLF. The z-score was limited to −2 to avoid representing variation based solely on the imputation strategy and not rooted in the data set. See Supplementary Table 2 for a list of all proteins present only in one group. nrPHLF, non-recovering posthepatectomy liver failure; rPHLF, recovering posthepatectomy liver failure.

The heat map revealed that five proteins linked to lipid metabolism were among the 20 most downregulated proteins in the rPHLF group (Figure 4). Two of these proteins (Nceh1 and Abhd2) play a role in lipolysis, two (Acaa1b and Echdc1) are involved in beta-oxidation of fatty acids, and one (Hmgcr) participates in lipogenesis. Among the 20 most upregulated proteins in the rPHLF group, one (Pla2g12b) is involved in lipid metabolism through lipolysis regulation and VLDL secretion.

### Hepatocyte Lipid Content

**Figure 5:**
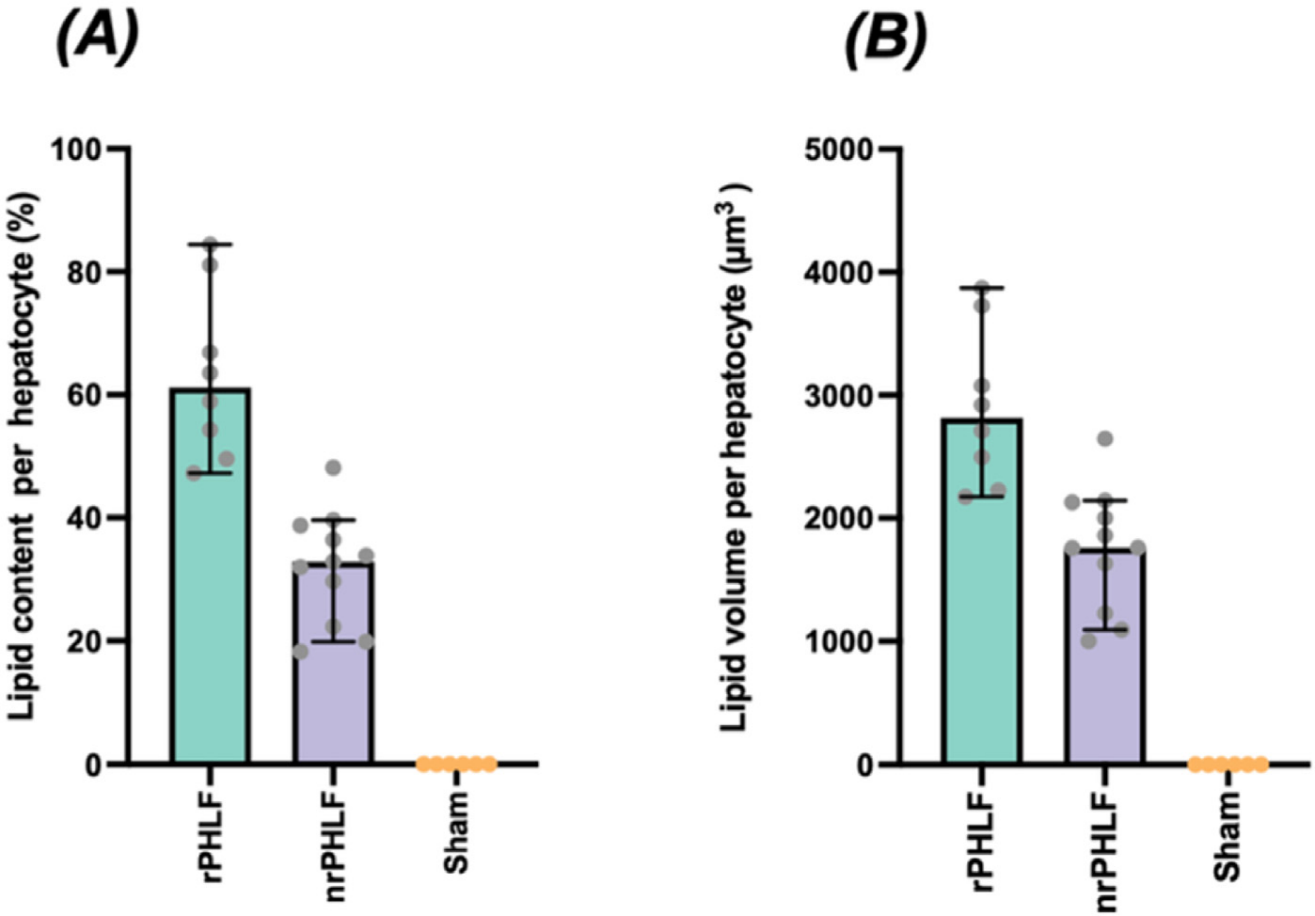
**Quantities of lipid droplets in the hepatocytes in rPHLF (n = 8), nrPHLF (n = 13), and sham (n = 6).** Each dot represents one animal. (A) The median lipid content per hepatocyte was estimated using a point grid on systematically uniformly and randomly selected fields of immunohistochemical stained liver sections, as described in the method section. (B) The median lipid volume per hepatocyte was calculated from the lipid content per hepatocyte and the previously estimated hepatocyte volume.^17^ nrPHLF, non-recovering posthepatectomy liver failure; rPHLF, recovering posthepatectomy liver failure.

The median hepatocyte lipid proportion was 1.6 times higher in rPHLF (61%; 95% CI: 48–82%) than in nrPHLF (32%; 95% CI: 22–39%), as shown in Figure 5.

Additionally, the median lipid volume in the hepatocytes of rPHLF was significantly higher (2208 μm^3^; 95% CI: 2208–3774 μm^3^) than that in nrPHLF (1759 μm^3^; 95% CI: 1188–2134 μm^3^). Lipid volume in the hepatocytes of the sham group was below the detection limit.

### Plasma Triglycerides

The mean triglyceride levels were 1.4 (95% CI: 0–3.4) mmol/l in the rPHLF group, 1.16 (95% CI: 1–1.4) mmol/l in the nrPHLF group, and 0.7 (95% CI: 0.6–0.9) mmol/l in the sham group. Although the mean triglyceride levels were higher in rats with rPHLF than in sham rats, these differences were not significant (*P* > 0.7).

## DISCUSSION

We conducted a comprehensive examination of liver tissues obtained from rats with rPHLF and nrPHLF after 90% PH. Our approach involved proteomic and stereological analyses aimed at investigating the protein expression and biological pathways involved in PHLF.

Enrichment analysis of proteins with significantly differential expression highlighted a possible critical role for lipid metabolism in the initial phase of liver regeneration and its potential contribution to averting nrPHLF. Increased lipid droplet accumulation was observed in rPHLF compared to that in nrPHLF.

Intracellular metabolic changes are vital during accelerated hepatocyte proliferation. First, cells require a sufficient supply of nutrients to facilitate proper functioning of intracellular processes. Second, for proliferation to occur, an adequate supply of essential building blocks including amino acids, nucleotides, carbohydrates, and lipids is crucial. This is in line with the findings from our stereological analyses of sections examined by light microscopy supported by electron microscopy, which showed that the rPHLF group exhibited a higher lipid content than the nrPHLF and sham groups. These findings indicate that lipids may play a crucial role in successful liver regeneration after major resection. This study did not allow us to determine whether their importance for successful regeneration is due to their function as fuel, building blocks, or both. Nevertheless, these findings prompted speculation regarding the potential efficacy of intravenous lipid administration for reversing PHLF. This necessitates further investigation, which we intend to address in a future porcine study.

Previous studies have suggested that lipid accumulation after extensive PH is associated with increased lipid uptake due to elevated blood flow to the liver remnant.^21^^,^^22^ Moreover, it has been proposed that the liver's secretory capacity is linearly correlated with the size of the liver, which is supported by increased secretory function when the liver remnant starts to regenerate.^5^ Our study results support these findings. First, we found that the low-density lipoprotein receptor, which is responsible for lipid uptake in hepatocytes, was upregulated in rPHLF compared to that in shams. Second, we found that HNF4A, a protein essential for lipoprotein metabolism and VLDL release from the liver into the bloodstream,^23^ was downregulated in the PH group compared to the sham group and in the rPHLF group compared to nrPHLF. Altogether, these findings suggest that the increased lipid accumulation observed in hepatocytes after major resection is due to increased lipid uptake and decreased secretion.

Lipid droplet development has been suggested to be an essential precursor of the proliferation response during liver regeneration after extensive resection, reaching its peak in the premitotic phase.^11^^,^^12^^,^^24^^,^^25^ Our enrichment analysis did not identify proliferation pathways that were differentially expressed between rPHLF and nrPHLF. Enrichment analysis of the sham group was beyond the scope of this study. However, HNF4A, which, among other functions, serves as a regulator of hepatocyte division by inhibiting hepatocyte proliferation,^26^ was downregulated in rPHLF compared to that in nrPHLF. In a previous study, we demonstrated that the proliferation response in the rPHLF group was evident 48 h after 90% PH, as judged by unbiased stereological methods. Conversely, the nrPHLF group exhibited impaired liver regeneration capacity and did not attain the peak point of proliferation.^17^ These findings, combined with the results obtained in this study, which revealed increased lipid accumulation and downregulated lipid metabolism in rPHLF, suggest that there may be an association between the regulation of lipid metabolism and a drive toward proliferation in the premitotic phase. In other words, during the critical 24-h period following extensive PH, the metabolic and regenerative demands of the liver may be prioritized over maintaining body homeostasis. Our observations are consistent with previous research indicating coordinated changes in specific lipid metabolic pathways during the transition of hepatocytes to a proliferative state.^27^

Several studies investigating preoperative hepatic steatosis have suggested that steatosis impair liver regeneration after resection.^8^^,^^28^ Contrary to these findings, our study revealed a significant elevation in hepatic lipid content in rPHLF compared to that in nrPHLF, suggesting a potential role of post-PH transitory steatosis in preventing nrPHLF. Consequently, our findings do not substantiate the assumption that lipid accumulation necessary is a marker of impaired regeneration after extensive liver resection. However, in the later stages of the proliferation response, when nrPHLF has been avoided, the liver appears to shift its focus toward retaining its function as the body's central organ for metabolic homeostasis, including the regulation of lipid metabolism. The liver's ability to prioritize metabolic tasks may be a crucial distinguishing factor between livers with preoperative steatosis and those without. This suggests that the beneficial effects of lipid accumulation may not solely arise from the function of the lipids but rather that the modified response likely reflects the liver's ability to effectively prioritize its limited resources in the critical phase after major resection.

Proteomics is a powerful tool for detecting, characterizing, and measuring protein expression within cells under various conditions. We used proteomics to gain insights into the complex biological processes involved in PHLF. Our metabolic findings were validated by examining adaptive changes in hepatic lipid content using stereological methods. Unlike qualitative or semiquantitative histological approaches, these methods allow the precise quantification of three-dimensional structures based on two-dimensional samples in a randomized and unbiased manner. Additionally, our light microscopy evaluations were confirmed using transmission electron microscopy.

Despite close and frequent observations using a quantitative scoring system to detect nrPHLF at least four times a day, some rats in the rPHLF group could have developed nrPHLF later, and some in the nrPHLF group may have recovered had they not been euthanized. Nonetheless, we previously identified the initial 48 h following 90% PH as the most critical period for the development of nrPHLF in rats;^29^ thus, we expected the majority of nrPHLF cases to be present within this time frame. Considering the significant difference in the physical condition of rPHLF and nrPHLF groups at 24 h after PH,^15^ wherein the nrPHLF group reached a level deemed ethically unacceptable to persist, and the fact that we previously observed fatal PHLF in rats to be detectable after 18 h, we are confident that all rats were appropriately assigned to the respective groups.

Prudence should be exercised when extrapolating the findings from rats to humans. However, the mechanisms underlying liver regeneration appear to be similar across mammals, with differences primarily observed in the dynamics of the processes involved.

In conclusion, we found a correlation between early liver regeneration, nrPHLF prevention, and protein downregulation intricately linked to lipid metabolism, cellular transport mechanisms, VLDL secretion, and hepatocyte proliferation. This response leads to an excessive intracellular lipid accumulation in the liver. Our findings suggest that transient hepatic steatosis after major liver resection is a promising prognostic marker for early liver regeneration and nrPHLF. This finding may be of therapeutic relevance in future studies.

## Credit authorship contribution statement

Lund A, Knudsen AR, Andersen KJ, Meier M, and Mortensen FV performed study the conception and design. Data collection was performed by Lund A. Analyses and interpretation of data were performed by Lund A, Thomsen M, Kirkegård J, Knudsen AR, Nyengaard JR, and Mortensen FV. The manuscript was drafted by Lund A. Critical revision and approval of the final paper was performed by Lund A, Thomsen M, Kirkegård J, Knudsen AR, Andersen KJ, Meier M, Nyengaard JR, and Mortensen FV.

## Ethics approval

This study is approved by the Danish National Committee for the Protection of Animals used for Scientific Purposes, Copenhagen, Denmark, under the license number 2021-15-0201-00978.

## Declaration of competing interest

The authors declare that they have no known competing financial interests or personal relationships that could have appeared to influence the work reported in this paper.
