# Supplementary material for: Role of Steatosis in Preventing Post-hepatectomy Liver Failure After Major Resection: Findings From an Animal Study
Source: J Clin Exp Hepatol. 2024 Nov 13;15(2):102453. doi: 10.1016/j.jceh.2024.102453 (PMC11652769; doi:10.1016/j.jceh.2024.102453)
Supplement: Multimedia component 1 [file mmc1.docx]

**Supplementary**

**Supplementary Table 1.** **Functional enrichment analysis based on significantly downregulated proteins in the recovering rats compared to the PHLF rats.**

| **Accession** | **Present in** | **Gene-name** |
| --- | --- | --- |
| Q9Z122 | nrPHLF | Fads2 |
| B2RZ77 | nrPHLF | Dpt |
| F7FNS3 | nrPHLF | Derl1 |
| Q01986 | nrPHLF | Map2k1 |
| Q63135 | nrPHLF | Cr1l |
| Q8CFD0 | nrPHLF | Sfxn5 |
| O55004 | nrPHLF | Rnase4 |
| A0A0G2K337 | nrPHLF | Abhd2 |
| Q9EQP5 | nrPHLF | Prelp |
| F1LNH3 | nrPHLF | Col6a2 |
| D3ZJR7 | nrPHLF | Dsc2 |
| Q63190 | nrPHLF | Emd |
| P51639 | nrPHLF | Hmgcr |
| Q9Z1M9 | nrPHLF | Smc1a |
| Q80W92 | nrPHLF | Vac14 |
| Q5U1W8 | nrPHLF | LOC100911295 |
| P51652 | nrPHLF | Akr1c18 |
| P82471 | nrPHLF | Gnaq |
| P53676 | nrPHLF | Ap3m1 |
| Q6EV70 | nrPHLF | Pofut1 |
| Q6AYG5 | nrPHLF | Echdc1 |
| P07871 | nrPHLF | Acaa1b |
| P37199 | nrPHLF | Nup155 |
| P29826 | nrPHLF | RT1-Bb |
| D4A7X5 | nrPHLF | Ppm1k |
| P22449 | nrPHLF | Hnf4a |
| Q5BK77 | nrPHLF | Rarres2 |
| Q675A5 | nrPHLF | Pla2g15 |
| A0A0G2K7M2 | nrPHLF | Rad23a |
| F1LY38 | nrPHLF | Cwc22 |
| D4A626 | nrPHLF | Clmn |
| Q5RK00 | nrPHLF | Mrpl46 |
| Q7TSU1 | nrPHLF | Arfgef2 |
| Q64620 | nrPHLF | Ppp6c |
| P29288 | nrPHLF | Acp5 |
| Q641X3 | nrPHLF | Hexa |
| Q63483 | nrPHLF | Rab38 |
| B2GV54 | nrPHLF | Nceh1 |
| P80349 | nrPHLF | N/A |
| P07323 | nrPHLF | Eno2 |
| P49793 | nrPHLF | Nup98 |
| D3Z863 | nrPHLF | Cwf19l1 |
| F1M1B3 | nrPHLF | Washc5 |
| F1M8F5 | nrPHLF | LOC100909605 |
| P42893 | nrPHLF | Ece1 |
| D4A3K5 | rPHLF | H1-1 |
| Q62751 | rPHLF | Ireb2 |
| M0RCI5 | rPHLF | Pla2g12b |
| Q8R500 | rPHLF | Mfn2 |
| D3ZDI7 | rPHLF | Ppp2r5a |
| Q63065 | rPHLF | Pdk1 |
| A0A0G2JY73 | rPHLF | Eif4g3 |
| B0K017 | rPHLF | Adprhl2 |
| P36876 | rPHLF | Ppp2r2a |
| Q5XIM0 | rPHLF | Bcs1l |
| Q8CGV7 | rPHLF | Thtpa |
| E9PTV9 | rPHLF | N/A |
| P24389 | rPHLF | Dio1 |
| F1M1E4 | rPHLF | Pnkd |
| F7ES73 | rPHLF | Nub1 |
| P58405 | rPHLF | Strn3 |
| F1LYX9 | rPHLF | Dsg2 |
| P21743 | rPHLF | Igfbp1 |
| D4A777 | rPHLF | Fam114a1 |
| D3Z9P1 | rPHLF | Kdsr |
| Q5XI64 | rPHLF | Abhd6 |
| Q56R17 | rPHLF | Kpna4 |
| D3ZYU4 | rPHLF | Cox20 |
| A0A0G2K1L0 | rPHLF | Tnc |
| Q9WTT2 | rPHLF | Clpb |
| E9PU01 | rPHLF | Chd4 |
| D4ADC2 | rPHLF | Helz2 |
| A0A0G2KAE1 | rPHLF | Lims2 |
| D4A8K4 | rPHLF | Tmem82 |
| D3ZJB7 | rPHLF | Slc30a10 |
| B0BNL2 | rPHLF | Pin1 |
| O54698 | rPHLF | Slc29a1 |
| Q3KRE3 | rPHLF | Gng10 |
| Q8CFM6 | rPHLF | Stab2 |
| P83953 | rPHLF | Kpna1 |
| D3ZR49 | rPHLF | Man1a2 |
| F1M446 | rPHLF | Ecpas |
| Q923V4 | rPHLF | Fbxo6 |
| Q569B7 | rPHLF | Rwdd4 |
| O08815 | rPHLF | Slk |
| D4A2H8 | rPHLF | Nudt12 |
| Q10728 | rPHLF | Ppp1r12a |
| D3ZFP4 | rPHLF | Mcm3 |
| Q9WUC8 | rPHLF | Plrg1 |
| D3ZRX9 | rPHLF | Cnn2 |
| P69682 | rPHLF | Necap1 |
| D4A5I9 | rPHLF | Myo6 |
| Q68FS1 | rPHLF | Nubp2 |
| Q99M63 | rPHLF | Smu1 |
| D4A7J8 | rPHLF | Prpf4 |
| D3ZJE0 | rPHLF | Edem1 |
| Q95II0 | rPHLF | RT1-CE5 |
| F1LRK4 | rPHLF | Grsf1 |
| G3V6X1 | rPHLF | Fbln2 |
| D3ZPL1 | rPHLF | Cpsf6 |
| F1LTN6 | rPHLF | N/A |
| D4A1B8 | rPHLF | Dctn3 |
| Q5PPJ4 | rPHLF | Dohh |
| G3V817 | rPHLF | Xrcc5 |
| A0A0G2JXF0 | rPHLF | N/A |
| G3V7P2 | rPHLF | Fgl2 |
| P16391 | rPHLF | N/A |
| Q01062 | rPHLF | Pde2a |
| Q68FW7 | rPHLF | Tars2 |
| Q6AYK5 | rPHLF | Lyar |
| Q5RJP0 | rPHLF | Akr1b7 |
| P63045 | rPHLF | Vamp2 |
| F7FFR1 | rPHLF | Rars2 |
| B1WBW4 | rPHLF | Armc10 |
| D3ZTR4 | rPHLF | Sumf2 |

**Table 2. List of all proteins only detected in one group.**
